# Clinical and Echocardiographic Risk Factors Predict Late Recurrence after Radiofrequency Catheter Ablation of Atrial Fibrillation

**DOI:** 10.1038/s41598-019-43283-7

**Published:** 2019-05-03

**Authors:** Yun Gi Kim, Jong-Il Choi, Ki Yung Boo, Do Young Kim, Suk-Kyu Oh, Hee-Soon Park, Kwang-No Lee, Jaemin Shim, Jin Seok Kim, Sang Weon Park, Seong-Mi Park, Wan Joo Shim, Young-Hoon Kim

**Affiliations:** Division of Cardiology, Department of Internal Medicine, Korea University College of Medicine and Korea University Medical Center, Seoul, Republic of Korea

**Keywords:** Interventional cardiology, Cardiac device therapy

## Abstract

The benefits of radiofrequency catheter ablation (RFCA) for patients with atrial fibrillation (AF) significantly decrease with late recurrence (LR). We aimed to develop a scoring system to identify patients at high and low risk for LR following RFCA, based on a comprehensive evaluation of multiple risk factors for AF recurrence, including echocardiographic parameters. We studied 2,352 patients with AF undergoing first-time RFCA in a single institution. The LR-free survival rate up to 5 years was measured using a Kaplan-Meier analysis. The influence of clinical and echocardiographic parameters on LR was calculated with a Cox-regression analysis. Duration of AF ≥4 years (hazard ratio [HR] = 1.75; p < 0.001), non-paroxysmal AF (HR = 3.18; p < 0.001), and diabetes (HR = 1.34; p = 0.015) were associated with increased risk of LR. Left atrial (LA) diameter ≥45 mm (HR = 2.42; p < 0.001), E/e′ ≥ 10 (HR = 1.44; p < 0.001), dense SEC (HR = 3.30; p < 0.001), and decreased LA appendage flow velocity (≤40 cm/sec) (HR = 2.35; p < 0.001) were echocardiographic parameters associated with increased risk of LR following RFCA. The LR score based on the aforementioned risk factors could be used to predict LR (area under curve = 0.717) and to stratify the risk of LR (HR = 1.45 per 1 point increase in the score; p < 0.001). In conclusion, LR after RFCA is affected by multiple clinical and echocardiographic parameters. This study suggests that combining these multiple risk factors enables the identification of patients with AF at high or low risk for having arrhythmia recurrence.

## Introduction

Atrial fibrillation (AF) is a substantial global health burden, associated with impaired quality of life and increased risk of cardiovascular events^1–4^. Until Haissaguerre and his colleagues discovered that the pulmonary vein was the main trigger of AF and introduced radiofrequency catheter ablation (RFCA), AF was not considered a curable disease^5^. Twenty years after its introduction, RFCA is recommended for symptomatic and antiarrhythmic drug (AAD)-refractory paroxysmal AF^6^. Based on recent advancements in ablation results and techniques, RFCA is also a preferred choice for patients with symptomatic persistent AF^6,7^. With RFCA compared to AADs, a significant improvement in quality of life has been well documented in multiple studies and meta-analyses^1,8–11^. Recent studies have also suggested that RFCA reduces the risk of ischemic stroke in patients with AF^12–14^. Furthermore, all-cause mortality may be significantly improved in selected patients, according to the CASTLE-AF trial^15^.

The benefits of RFCA are closely associated with late recurrence (LR)^1,16–18^. However, the success rate of RFCA, especially in patients with non-paroxysmal AF, is still not optimal despite recent improvements^7^. The reported success rate of RFCA is between 40–80%, depending on patient characteristics, definition of LR, repeat procedures, and follow-up duration^7,15,19^. Large left atrium (LA) size, non-paroxysmal AF, and extensive late gadolinium enhancement of LA are well-established risk factors for LR following RFCA^20–24^. Duration of AF, age, body mass index (BMI), and various transthoracic and transesophageal echocardiographic parameters might also predict LR after undergoing RFCA. Furthermore, repeat procedures have proven its efficacy in reducing the rate of LR^25,26^. Various studies have reported individual risk factors for AF recurrence, but there has been no comprehensive analysis of multiple risk factors for LR. Hence, we sought to develop a scoring system to identify the true high- and low-risk groups for LR following their last RFCA procedure, based on a comprehensive evaluation of multiple risk factors for AF recurrence.

## Methods

### Patients

We analyzed consecutive patients with AF who underwent their first RFCA at Korea University Anam Hospital from June 1998 and May 2016. All patients who underwent RFCA in our institution were included, and there were no specific exclusion criteria. The current study was approved by the Institutional Review Board of Korea University Anam Hospital, which ensured appropriate ethical and bioethical conduct. Informed consent was waived since this was a retrospective study. All patient records and medical information were anonymized prior to analysis. The protocol of the current study was consistent with the ethical guidelines of the 2008 Helsinki Declaration.

### Radiofrequency catheter ablation

Double trans-septal punctures were performed using a Brockenbrough needle and two SL1 sheaths. Three-dimensional mapping of LA was performed with either an EnSite NavX/Velocity (St. Jude Medical, St. Paul, MN, USA) or a CARTO (Biosense Webster, Irvine, CA, USA) system. Pulmonary vein isolation was performed for paroxysmal AF. After successful isolation of pulmonary veins, non-pulmonary vein trigger focus evaluation was performed by AF induction with rapid atrial pacing under isoproterenol infusion and subsequent direct current cardioversion. Additional non-pulmonary vein trigger ablation was performed if AF was initiated by trigger activity from non-pulmonary vein focus. End point of the procedure in paroxysmal AF patients was the absence of both pulmonary vein and non-pulmonary vein trigger focus. However, additional substrate modifications were performed in patients with paroxysmal AF if the operator considered non-inducibility more important than trigger focus elimination. Acquiring non-inducibility was the endpoint of the procedure for non-paroxysmal AF. Pulmonary vein isolation and additional complex fractionated atrial electrogram-guided or linear ablation were performed for non-paroxysmal AF, based on the operators’ discretion. Patients received routine anticoagulation treatment for 2–3 months after RFCA. Regular 12-lead surface electrocardiography and Holter evaluation was performed at 3, 6, 9, and 12 months after discharge. If patient complained of symptoms suggestive of recurrence but had no evidence of AF or atrial tachycardia in 12-lead surface electrocardiography or Holter evaluation, event recorder evaluation was performed.

### Echocardiography

Both transthoracic echocardiography (TTE) and transesophageal echocardiography (TEE) were performed before RFCA. All echocardiographic evaluations were performed by cardiologists dedicated in the field of cardiac imaging. During TTE, LA size, ejection fraction of left ventricle (LV), mitral valve inflow velocity (E), and mitral annular tissue velocity (E′) were measured. Multiple views (high esophageal 0°, 45°, 60°, and 120° views) were obtained during TEE evaluation. Emptying (forward), filling (backward), and the average flow velocity of the LA appendage (LAA) were measured. The presence of spontaneous echocontrast (SEC) and thrombus in LA or LAA were carefully evaluated. SEC was divided into grades of very mild (minimal echogenicity, only detectable transiently, or increasing gain setting required for the detection), mild (detectable without increasing gain setting), moderate (dense, swirling echogenic material; echogenic signal is dense in LAA compared to LA), or severe (dense, swirling echogenic material; echogenic signal is equivocal in LAA and LA). Dense SEC was defined as a composite of moderate and severe SEC.

### Definitions

In the current study, LR was defined as any atrial tachyarrhythmia lasting for more than 30 seconds in Holter monitoring or in the event recorder after the 3-month blanking period. Single strip (10 seconds) of 12-lead surface electrocardiography showing any type of atrial tachyarrhythmia was also defined as LR. The LR-free survival rate was calculated for both 5 years of follow-up and for the full follow-up period. Paroxysmal AF was defined as AF lasting for less than 7 days, and non-paroxysmal AF as AF lasting for more than 7 days. The duration of AF was calculated by the difference between the date of initial symptom onset (or first diagnosis of AF) and the RFCA index date. The LR score was calculated using the multiple risk factors for LR identified in the current study. The LR score was utilized to predict and stratify the risk of LR after the first and last RFCA procedures.

### Statistical analysis

Continuous variables are described as means ± standard deviations, and were compared using a Student’s t-test. Categorical variables are presented as percentile values, and were compared with a chi-square test or Fisher’s exact test as appropriate. LR-free survival was measured by Kaplan-Meier survival curve analysis, and the difference between groups was assessed using a log-rank test. Cox regression analysis was performed to calculate the hazard ratio (HR) and its 95% confidence interval (CI) for each risk factor. Receiver operating characteristic (ROC) curve analysis with a calculation of the area under curve (AUC) was performed to evaluate the efficacy of the LR score to predict LR. Missing data were excluded from each analysis, and no imputation was performed. All significance tests were two-tailed, and p values equal or less than 0.05 were considered statistically significant. All statistical analyses were performed with SPSS version 21.0 (IBM, Armonk, NY, USA).

## Results

### Patients

A total of 2,352 patients with AF who underwent RFCA for the first time were studied. The number of procedures was 2,997, with 546 patients having two procedures; 83, three; 12, four; 3, five; and 1, six procedures. The mean number of procedures per patient was 1.27, and the mean time interval between the first-time RFCA and the repeat procedure was 849 days. The baseline characteristics of the study population are summarized in Supplementary Table S1. The mean age was 55.4 ± 10.9 years, and 79.6% were male. Non-paroxysmal AF was diagnosed in 40.2% of the patients. The mean LA diameter and CHA_2_DS_2_-VASc score were 41.1 ± 6.0 mm and 1.3 ± 1.3 points, respectively. The total follow-up duration was 10,023 patient * years. During the follow-up, 969 (41.2%) and 613 (26.1%) patients experienced LR after their first and last RFCA procedures, respectively.

### Risk factors for LR after last RFCA

Baseline clinical and echocardiographic characteristics were compared between patients with and without LR after their last RFCA (Table 1). Patients who experienced LR following the last RFCA were older (54.7 ± 10.9 vs. 57.3 ± 10.8 years old; p < 0.001) and had a high BMI (24.8 ± 2.9 vs. 25.2 ± 3.3 kg/m^2^; p = 0.014); long AF duration (4.4 ± 4.3 vs. 5.9 ± 5.4 years; p < 0.001); high CHA_2_DS_2_-VASc score (1.2 ± 1.2 vs. 1.6 ± 1.4; p < 0.001); large LA (40.3 ± 5.6 vs. 43.5 ± 6.3 mm; p < 0.001); low LV ejection fraction (55.1 ± 5.9 vs. 54.1 ± 6.7%; p = 0.001); high E/E′ (8.7 ± 3.3 vs. 9.6 ± 5.6; p = 0.001); and low average LAA flow velocity (51.3 ± 20.7 vs. 41.0 ± 19.9 cm/sec; p < 0.001). They also had a higher proportion of female sex (19.4% vs. 23.2%; p = 0.049), non-paroxysmal AF (32.9% vs. 60.7%; p < 0.001), SEC (16.5% vs. 35.3%; p < 0.001), and dense SEC (2.0% vs. 8.3%; p < 0.001). Among these parameters, BMI and sex status were not associated with an increased risk of LR in Cox-regression or ROC curve analyses, and were not included in the subsequent analysis. Among individual component of CHA_2_DS_2_-VASc score, diabetes, heart failure, and previous history of stroke or TIA were associated with increased risk of LR. However, heart failure and previous history of stroke or TIA were not included in the subsequent analysis since LV ejection fraction, LAA flow velocity, and presence of dense SEC were included instead.

**Table 1:** Baseline characteristics of patients with and without LR after last RFCA.

	LR (−) (n = 1,739)	LR (+) (n = 613)	p value
Age (years old)	54.7 ± 10.9	57.3 ± 10.8	<0.001
Male sex	1,401 (80.6%)	471 (76.8%)	0.049
Body weight (kg)	70.6 ± 11.0	70.9 ± 11.7	0.634
Height (cm)	168.3 ± 8.2	167.4 ± 8.4	0.014
BMI (kg/m^2^)	24.8 ± 2.9	25.2 ± 3.3	0.014
Non-paroxysmal AF	573 (32.9%)	372 (60.7%)	<0.001
AF duration (years)	4.4 ± 4.3	5.9 ± 5.4	<0.001
Heart failure	116 (6.7%)	64 (10.4%)	0.003
Hypertension	615 (35.4%)	250 (40.8%)	0.017
Diabetes mellitus	173 (9.9%)	86 (14.0%)	0.006
Previous CVA, TIA, or embolism	123 (7.1%)	62 (10.1%)	0.016
Vascular disease	138 (7.9%)	82 (13.4%)	<0.001
CHA_2_DS_2_-VASc score	1.2 ± 1.2	1.6 ± 1.4	<0.001
TTE findings			
LA diameter (mm)	40.3 ± 5.6	43.5 ± 6.3	<0.001
LV ejection fraction (%)	55.1 ± 5.9	54.1 ± 6.7	0.001
E	65.1 ± 16.8	68.1 ± 17.4	0.001
E′	8.1 ± 2.4	7.9 ± 2.4	0.137
E over E′	8.7 ± 3.3	9.6 ± 5.6	0.001
TEE findings			
LAA emptying velocity (cm/sec)	50.4 ± 21.7	39.9 ± 20.4	<0.001
LAA filling velocity (cm/sec)	52.1 ± 22.1	42.0 ± 21.2	<0.001
LAA average velocity (cm/sec)	51.3 ± 20.7	41.0 ± 19.9	<0.001
SEC	259 (16.5%)	197 (35.3%)	<0.001
Dense SEC	31 (2.0%)	46 (8.3%)	<0.001
Thrombus	2 (0.1%)	3 (0.5%)	0.117
Laboratory findings			
Hemoglobin (g/dL)	14.7 ± 1.4	14.5 ± 1.6	0.020
WBC (10^3^/μL)	6.5 ± 3.8	6.4 ± 1.7	0.474
Platelets (10^3^/μL)	209.4 ± 49.2	202.3 ± 48.9	0.002
Serum creatinine (mg/dL)	1.0 ± 0.3	1.1 ± 0.5	0.040

*Results are presented as n (%) or means with standard deviations.

AF: atrial fibrillation; BMI: body mass index; CVA: cerebrovascular accident; LA: left atrium; LAA: left atrial appendage; LR: late recurrence; LV: left ventricle; RFCA: radiofrequency catheter ablation; SEC: spontaneous echocontrast; TEE: transesophageal echocardiography; TIA: transient ischemic attack; TTE: transthoracic echocardiography; WBC: white blood cell.

**Figure 1:**
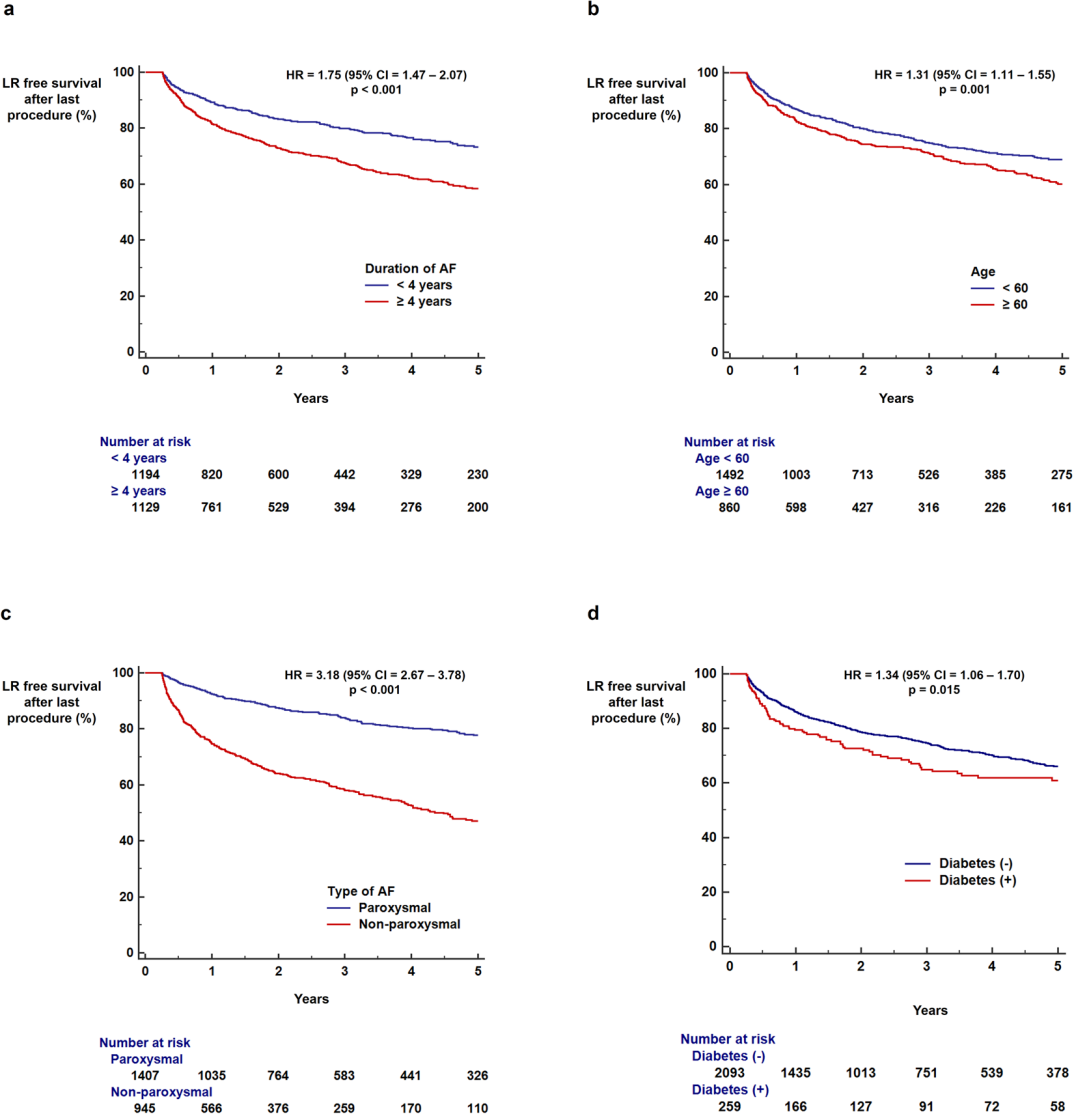
Influence of clinical parameters on LR. Kaplan-Meier curve analysis of cumulative incidence of LR for up to 5 years following the last RFCA, according to AF duration (**a**) age (**b**) AF type (**c**) and diabetes (**d**). AF: atrial fibrillation; CI: confidence interval; HR: hazard ratio; LR: late recurrence; RFCA: radiofrequency catheter ablation.

Patients with AF duration ≥4 years (73.3% vs. 58.3%, log-rank p < 0.001; HR = 1.75, 95% CI = 1.47–2.07, p < 0.001; Fig. 1a), age ≥60 years (69.0% vs. 60.1%, log-rank p = 0.001; HR = 1.31, 95% CI = 1.11–1.55, p = 0.001; Fig. 1b), non-paroxysmal AF (77.7% vs. 47.0%, log-rank p < 0.001; HR = 3.18, 95% CI = 2.67–3.78, p < 0.001; Fig. 1c), and diabetes (66.1% vs. 60.8%, log-rank p = 0.015; HR = 1.34, 95% CI = 1.06–1.70, p = 0.015; Fig. 1d) showed a significantly lower rate of LR-free survival for up to 5 years following the last RFCA. Kaplan-Meier curves for the full follow-up period are depicted in Supplementary Fig. S1.

**Figure 2:**
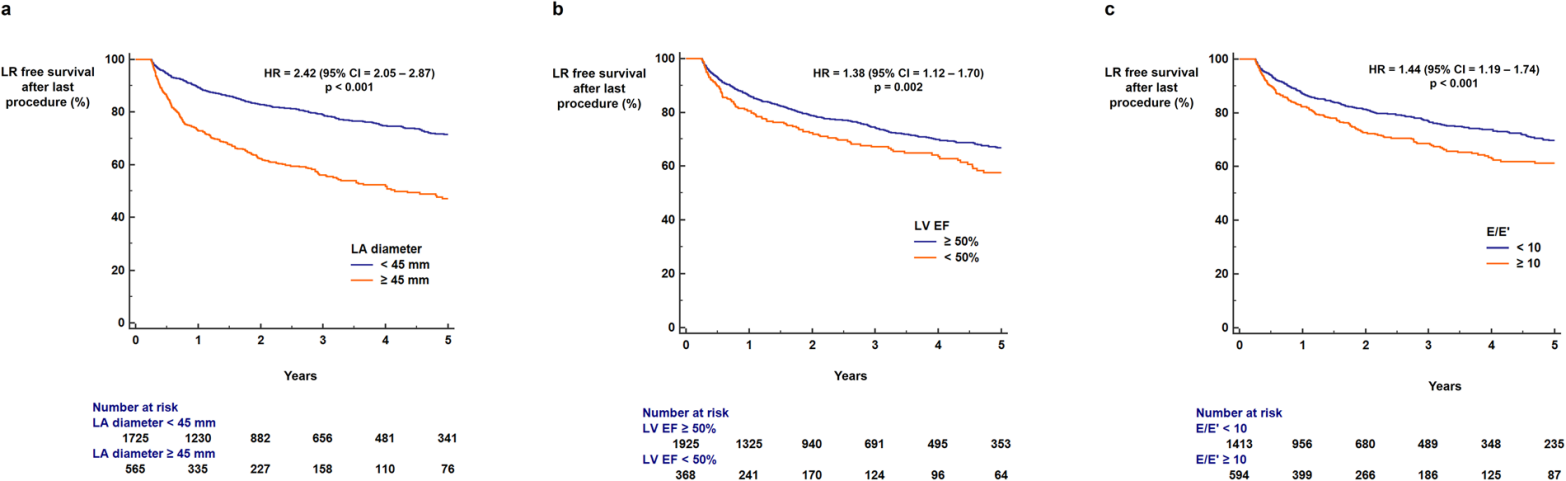
TTE risk factors for LR. LA diameter ≥45.0 mm (**a**) LV EF <50% (**b**) and E/E′ ≥ 10 (**c**) were significantly associated with increased risk of LR following the last RFCA. CI: confidence interval; HR: hazard ratio; LA: left atrium; LR: late recurrence; LV: left ventricle; RFCA: radiofrequency catheter ablation; TTE: transthoracic echocardiography.

**Figure 3:**
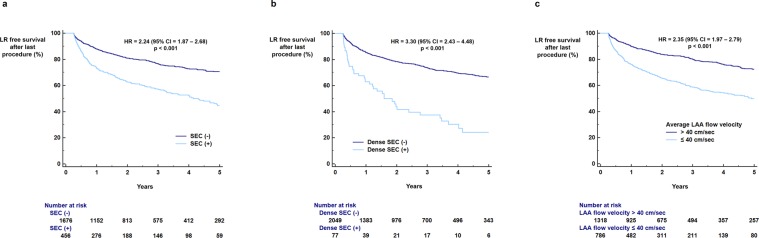
TEE risk factors for LR. The presence of SEC (**a**) dense SEC (**b**) and decreased LAA flow velocity (**c**) were significantly associated with increased risk of LR following the last RFCA. CI: confidence interval; HR: hazard ratio; LAA: left atrial appendage; LR: late recurrence; RFCA: radiofrequency catheter ablation; SEC: spontaneous echocontrast; TEE: trans-esophageal echocardiography.

LA diameter ≥45 mm (71.5% vs. 47.0%, log-rank p < 0.001; HR = 2.42, 95% CI = 2.05–2.87, p < 0.001; Fig. 2a), LV ejection fraction < 50% (66.7% vs. 57.4%, log-rank p = 0.002; HR = 1.38, 95% CI = 1.12–1.70, p = 0.002; Fig. 2b), and E/E′ ≥ 10 (69.7% vs. 61.2%, log-rank p < 0.001; HR = 1.44, 95% CI = 1.19–1.74, p < 0.001; Fig. 2c) were also associated with a significantly increased risk of AF recurrence for up to 5 years after the last RFCA procedure. The presence of SEC (70.7% vs. 44.6%, log-rank p < 0.001; HR = 2.24, 95% CI = 1.87–2.68, p < 0.001; Fig. 3a), dense SEC (66.6% vs. 24.2%, log-rank p < 0.001; HR = 3.30, 95% CI = 2.43–4.48, p < 0.001; Fig. 3b), and decreased average LAA flow velocity (≤40 cm/sec) (72.5% vs. 49.7%, log-rank p < 0.001; HR = 2.35, 95% CI = 1.97–2.79, p < 0.001; Fig. 3c) found during TEE evaluation showed strong associations with LR for up to 5 years after the last RFCA. Kaplan-Meier curves of the full follow-up duration for TTE and TEE risk factors are presented in Supplementary Figs S2 and S3, respectively.

### LR score

We created the LR score to identify patients who are at high or low risk for LR after their last RFCA. Multivariate Cox regression analysis was performed for individual risk factors identified in the previous analysis (Figs 1–3) and the results are summarized in Table 2. Based on the multivariate analysis, factors with p value less than 0.1 was selected to be included in the LR scoring system.

**Table 2:** Univariate and multivariate analysis of risk factors for LR after last RFCA.

	Univariate			Multivariate		
	HR	95% CI	p value	HR	95% CI	p value
Age (per 1 year)	1.016	1.008–1.024	<0.001	0.999	0.988–1.010	0.842
Non-paroxysmal AF	3.177	2.674–3.775	<0.001	2.012	1.591–2.546	<0.001
Duration of AF (per 1 year)	1.045	1.031–1.059	<0.001	1.036	1.018–1.055	<0.001
LA diameter (per 1 mm)	1.093	1.078–1.108	<0.001	1.040	1.020–1.060	<0.001
LV EF (per 1%)	0.978	0.966–0.989	<0.001	1.009	0.993–1.026	0.250
E/e′ (per 1)	1.051	1.035–1.068	<0.001	1.031	1.008–1.054	0.007
LAA flow velocity (per 1 cm/sec)	0.976	0.972–0.980	<0.001	0.988	0.982–0.994	<0.001
Dense SEC	3.297	2.427–4.479	<0.001	1.437	0.975–2.118	0.067
Diabetes mellitus	1.343	1.058–1.703	0.015	1.431	1.096–1.869	0.008

AF: atrial fibrillation; CI: confidence interval; HR: hazard ratio; LA: left atrium; LAA: left atrial appendage; LR: late recurrence; LV EF: left ventricular ejection fraction; RFCA: radiofrequency catheter ablation; SEC: spontaneous echocontrast.

**Figure 4:**
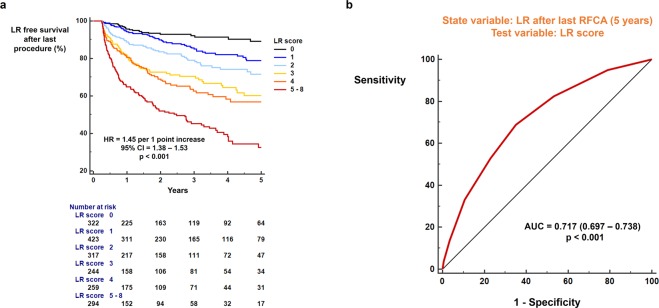
Predictive value of the LR score. (**a**) Risk of LR stratified by LR score. (**b**) ROC curve analysis of the LR score which showed significant predictive value. AUC: area under curve; CI: confidence interval; HR: hazard ratio; LR: late recurrence; RFCA: radiofrequency catheter ablation; ROC: receiver operating characteristic.

Each risk factor was assigned points as follows: one point for AF duration ≥4 years, LA diameter ≥45 mm, E/e′ ≥ 10, LAA flow velocity ≤40 cm/sec, presence of dense SEC, history of diabetes; two points for non-paroxysmal AF. The risk of LR differed significantly according to LR score (HR = 1.45 per 1 point increase, 95% CI = 1.38–1.53; p < 0.001; Fig. 4a). ROC curve analysis also revealed that the LR score could predict LR after the last RFCA (AUC = 0.717; 95% CI = 0.697–0.738; p < 0.001; Fig. 4b). Patients with LR score 0 showed excellent results, with 89.1% of the patients maintaining sinus rhythm for up to 5 years of follow up. However, patients with LR score ≥5 showed poor prognosis, with only 32.6% of the patients having LR-free survival through 5 years of follow up. Late recurrence following first RFCA can also be predicted by LR score (HR = 1.34 per 1 point increase, 95% CI = 1.28–1.39, p < 0.001; AUC = 0.687, p < 0.001; Supplementary Fig. S4).

The CHA_2_DS_2_-VASc score also had predictive value for LR after last RFCA (HR = 1.17 per 1 point increase; 95% CI = 1.11–1.25; p < 0.001; Supplementary Fig. S5a). However, the LR score system significantly outperformed the CHA_2_DS_2_-VASc score (AUC = 0.717 vs. 0.571; p < 0.001; Supplementary Fig. S5b).

### LR after first and last RFCA

**Figure 5:**
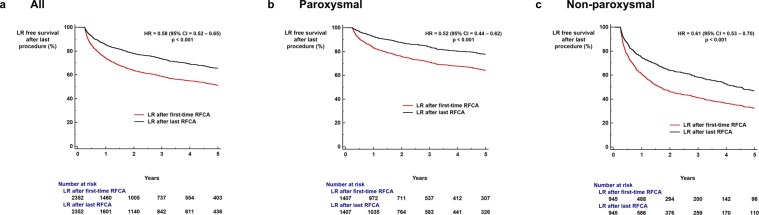
Impact of repeat procedures. Repeat procedures were associated with improved outcomes in patients with AF undergoing RFCA (**a**) regardless of AF type (**b**,**c**). AF: atrial fibrillation; CI: confidence interval; RFCA: radiofrequency catheter ablation.

Undergoing repeat procedures was associated with significantly improved LR-free survival (65.6% vs. 51.2%, log-rank p < 0.001; HR = 0.58, 95% CI = 0.52–0.65, p < 0.001; Fig. 5a). The relationship of improved outcome with undergoing repeated procedures was maintained in both paroxysmal AF (77.7% vs. 64.0%, log-rank p < 0.001; HR = 0.52, 95% CI = 0.44–0.62, p < 0.001; Fig. 5b) and non-paroxysmal AF (47.0% vs. 32.4%, log-rank p < 0.001; HR = 0.61, 95% CI = 0.53–0.70, p < 0.001; Fig. 5c).

## Discussion

In the current study, multiple clinical and echocardiographic parameters were identified as risk factors for LR following RFCA. The clinical risk factors were type of AF, duration of AF, and diabetes. Left atrial diameter, E/e′, presence of dense SEC, and decreased LAA flow velocity were echocardiographic risk factors for LR following RFCA. The LR score, which was created based on the aforementioned risk factors, could be used to predict LR, with an AUC of 0.717. Patient groups based on LR score had significantly different risks for experiencing LR following RFCA. In current clinical practice, the type of AF and diameter of LA are usually taken into account when predicting the success rate of RFCA in patients with AF. However, our analysis revealed that recurrence after RFCA is affected by multiple other risk factors as well. A significant number of patients (91%) underwent both TTE and TEE evaluation, and we were able to examine multiple echocardiographic parameters that reflect the hemodynamics of LA, LAA, and LV.

### Clinical risk factors

In accordance with previous studies, we found that longer duration of AF (≥4 years) and history of diabetes mellitus were independently associated with a significantly increased risk of LR following the last RFCA. Non-paroxysmal AF was associated with a 3-fold increased risk of LR following the last RFCA. The CHA_2_DS_2_-VASc score system, which is composed of clinical factors, was able to predict LR after RFCA. However, the LR score system which is composed of both clinical and echocardiographic parameters, was significantly superior to the CHA_2_DS_2_-VASc score system for the prediction of LR.

Previous studies have demonstrated that AF is a progressive disease, and a substantial number of paroxysmal AF events eventually progress to non-paroxysmal AF as time goes on^27,28^. Progressive structural remodeling of LA is probably the underlying mechanism^28^. Therefore, it is not surprising that a longer AF duration is associated with a higher recurrence rate. The benefit of performing early RFCA in patients with paroxysmal AF before LA remodeling and conversion to non-paroxysmal AF should be tested in future clinical trials. Previous studies identified female sex as a risk factor for LR after RFCA^7,29,30^. However, in our analysis, female sex was not associated with increased risk of LR following the last RFCA in a Cox regression analysis. The role of sex status as a risk factor for recurrence after RFCA needs to be clarified in future studies.

### Echocardiographic risk factors

LA diameter is traditionally regarded as a risk factor for recurrence after RFCA. Our study also showed that large LA diameter was associated with a significantly increased risk of LR following RFCA. In addition, LV ejection fraction and E/e′ were related to an increased risk of LR after RFCA. Cardiac chambers are not independent structures, and both LV systolic and diastolic function affect the LA. Decreased LV function can increase LA filling pressure and eventually dilate the LA. An extreme result of diastolic dysfunction is hypertrophic cardiomyopathy, which is associated with both AF and ischemic stroke. Therefore, post-procedural care should not only focus on LA but also on LV.

We also identified TEE parameters that were associated with an increased risk of LR following the last RFCA. Previous studies focused on LA diameter as a risk factor for recurrence, but TEE parameters such as the presence of dense SEC and decreased flow velocity of the LAA also deserve further attention. The presence of SEC is generally considered to be a consequence of impaired blood flow, atrial fibrosis, and decreased contraction of the LA and the LAA^31^. Therefore, the presence of SEC in the LA and the LAA can be regarded as a surrogate marker for decreased LA and LAA hemodynamic function. Left atrial remodeling due to AF progression will result in more fibrosis in the LA, which will in turn decrease its contractility and will eventually result in development of SEC^31,32^. Therefore, SEC can be considered as a common final result of LA remodeling, fibrosis, dilation, and decreased hemodynamic function. In our study, SEC and dense SEC were associated with a 2.24-fold and a 3.30-fold increased risk of LR following the last RFCA, respectively. Decreased LAA flow velocity, which reflects decreased hemodynamic function of the LA and the LAA, was also associated with a 2.35-fold increased risk of LR. Physicians should consider these unfavorable TEE findings when deciding whether to perform RFCA.

### Identification of patients at low and high risk of LR following RFCA

The risk of LR differed significantly according to LR score with 45% increased risk of recurrence for every 1 point increase in LR score. The low-risk group with LR score 0 showed excellent clinical results, with 89.1% of them having no arrhythmia recurrence up to 5 years of follow up. These patients have the highest chance to maintain sinus rhythm after RFCA, and electrophysiologists should discuss these findings with their patients during decision making. Repeat procedures were shown to be effective in improving the LR-free survival rate. Patients with either paroxysmal or non-paroxysmal AF derived substantial benefit from repeat procedures and therefore, repeat procedures may be recommended to patients who experienced LR, regardless of AF type. Patients with high LR score (≥5), only 32.6% maintained sinus rhythm despite repeat procedures during 5 years of follow up. Patients with a high LR score can be informed that the success rate of RFCA, including repeat procedures, might not fulfill their expectations.

### Limitations

The current study has several shortcomings. First, this was a retrospective analysis. Second, magnetic resonance image data were not available from a substantial number of patients, and therefore were not included in the analysis. Third, we could not retrieve the rhythm status during TEE evaluation which might further increase the predictive value of LAA flow velocity. Integration of late gadolinium enhancement data into the scoring system might further improve the prognostic value of the LR score.

## Conclusions

This study demonstrated that the risk factors for LR following the last RFCA were longer duration of AF, non-paroxysmal AF, diabetes, large LA diameter, high E/e′, presence of dense SEC, and decreased flow velocity in the LAA. The LR scoring system based on the aforementioned risk factors can be used to predict future risk of arrhythmia recurrence following RFCA.

## Supplementary information

Supplementary information

## Data Availability

All data generated or analysed during this study are included in this published article (and its Supplementary Information Files).
