# Supplementary material for: Clinical and Echocardiographic Risk Factors Predict Late Recurrence after Radiofrequency Catheter Ablation of Atrial Fibrillation
Source: Sci Rep. 2019 May 3;9:6890. doi: 10.1038/s41598-019-43283-7 (PMC6499800; doi:10.1038/s41598-019-43283-7)
Supplement: Supplementary file 1 — Supplementary information [file 41598_2019_43283_MOESM1_ESM.pdf]

Clinical and Echocardiographic Risk Factors Predict Late Recurrence after Radiofrequency Catheter Ablation of Atrial Fibrillation

Yun Gi Kim, MD; Jong-Il Choi, MD, PhD, MHSc*; Ki Yung Boo, MD; Do Young Kim, MD; Suk-Kyu Oh, MD; Hee-Soon Park, MD; Kwang-No Lee, MD; Jaemin Shim, MD, PhD; Jin Seok Kim, MD, PhD; Sang Weon Park, MD; Seong-Mi Park, MD, PhD; Wan Joo Shim, MD, PhD; Young-Hoon Kim, MD, PhD.

Division of Cardiology, Department of Internal Medicine, Korea University College of Medicine and Korea University Medical Center, Seoul, Republic of Korea

*** Address for correspondence**

Jong-Il Choi, MD, PhD, MHSc

Division of Cardiology, Department of Internal Medicine, Korea University College of Medicine and Korea University Medical Center, Seoul, Republic of Korea

73 Inchon-ro, Seongbuk-gu, Seoul 02841, Republic of Korea.

Tel: 82-2-920-5445

Fax: 82-2-927-1478

E-mail: jongilchoi@korea.ac.kr

Brief title: Risk stratification of AF recurrence

Supplementary Table S1. Baseline characteristics of the study population.

	**Total population (N = 2,352)***
Clinical findings	
Age (years old)	55.4 ± 10.9
Male sex	1,872 (79.6%)
BMI (kg/m^2)	24.9 ± 3.0
Non-paroxysmal AF	945 (40.2%)
AF duration (year)	4.8 ± 4.7
Heart failure	180 (7.7%)
Hypertension	865 (36.8%)
Diabetes mellitus	259 (11.0%)
Previous CVA, TIA, or systemic embolism	185 (7.9%)
Vascular disease	220 (9.3%)
CHA_2DS_2-VASc score	1.3 ± 1.3
TTE findings	
LA diameter (mm)	41.1 ± 6.0
LV ejection fraction (%)	54.9 ± 6.2
E over E'	8.9 ± 4.0
TEE findings	
LAA average velocity (cm/sec)	48.6 ± 21.0
SEC	456 (21.4%)
Dense SEC	77 (3.6%)
Thrombus	5 (0.2%)
Laboratory findings	
WBC (10^3/μL)	6.5 ± 3.4
Hemoglobin (g/dL)	14.7 ± 1.4
Platelets (10^3/μL)	207.6 ± 49.2
Serum creatinine (mg/dL)	1.0 ± 0.4

*Results are presented as n (%) or means with standard deviations.

AF: atrial fibrillation; BMI: body mass index; CVA: cerebrovascular accident; LA: left atrium; LAA left atrial appendage; LV: left ventricle; SEC: spontaneous echocontrast; TEE: transesophageal echocardiography; TIA: transient ischemic attack; TTE: transthoracic echocardiography; WBC: white blood cells.

Supplementary figure legends

Figure S1

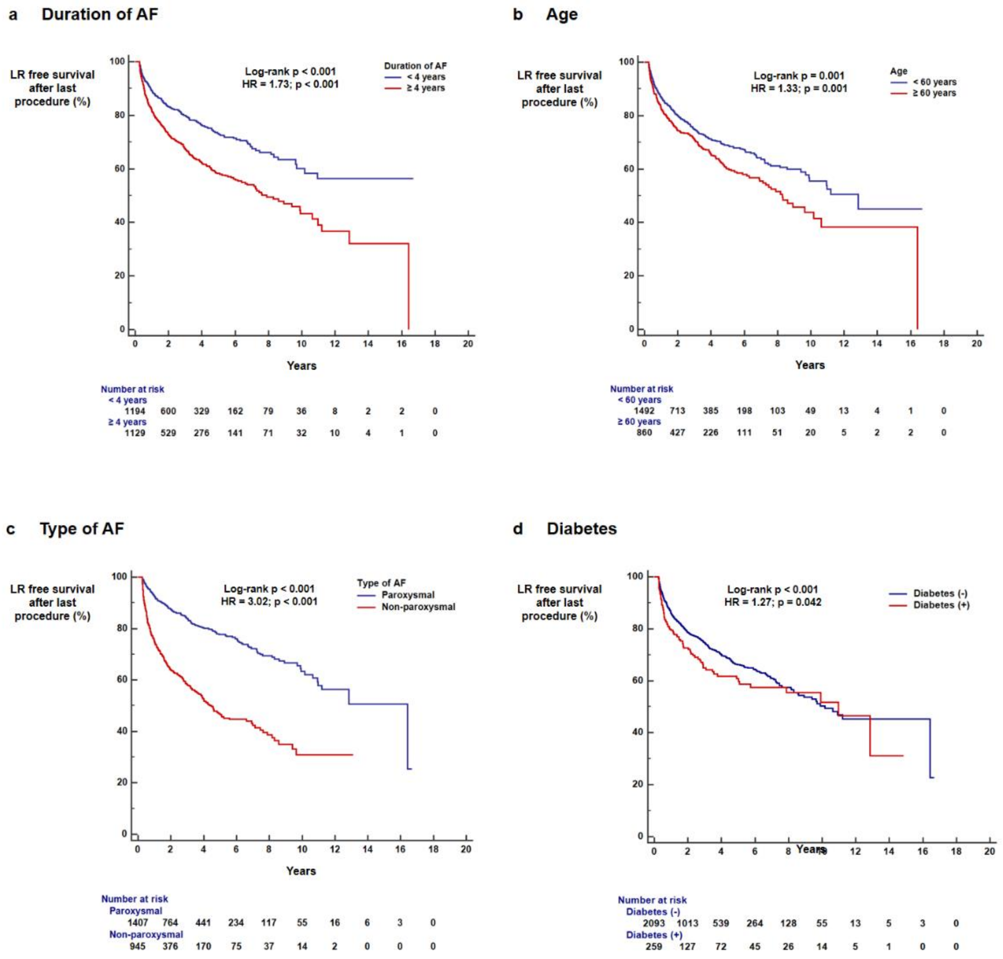

Supplementary Figure S1. Influence of clinical parameters on LR.

Kaplan-Meier curve analysis of the cumulative incidence of LR following the last RFCA for the full follow-up duration according to AF duration **(a)**, age **(b)**, AF type **(c)**, and diabetes **(d)**.

AF: atrial fibrillation; HR: hazard ratio; LR: late recurrence; RFCA: radiofrequency catheter ablation.

Figure S2

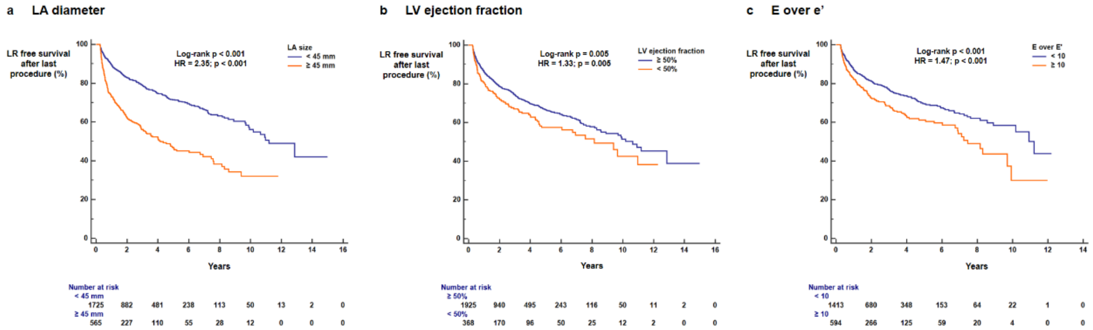

Supplementary Figure S2. TTE risk factors for LR.

LA diameter ≥ 45.0 mm **(a),** LV EF < 50% **(b)**, and E/E' ≥ 10 **(c)** were significantly associated with increased risk of LR following the last RFCA (full follow-up duration).

HR: hazard ratio; LA: left atrium; LR: late recurrence; LV: left ventricle; RFCA: radiofrequency catheter ablation; TTE: trans-thoracic echocardiography.

Figure S3

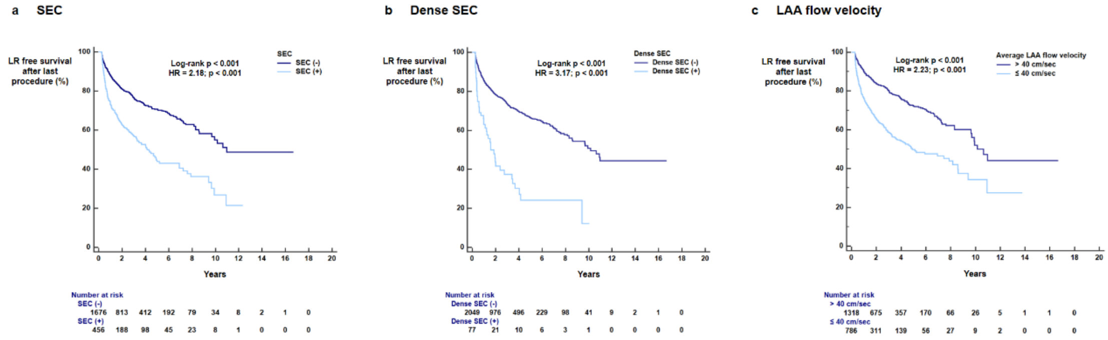

Supplementary Figure S3. TEE risk factors for LR.

Presence of SEC **(a)**, dense SEC **(b),** and decreased LAA flow velocity **(c)** were significantly associated with increased risk of LR following the last RFCA (full follow-up duration).

HR: hazard ratio; LAA: left atrial appendage; LR: late recurrence; RFCA: radiofrequency catheter ablation; SEC: spontaneous echocontrast; TEE: trans-esophageal echocardiography.

Figure S4

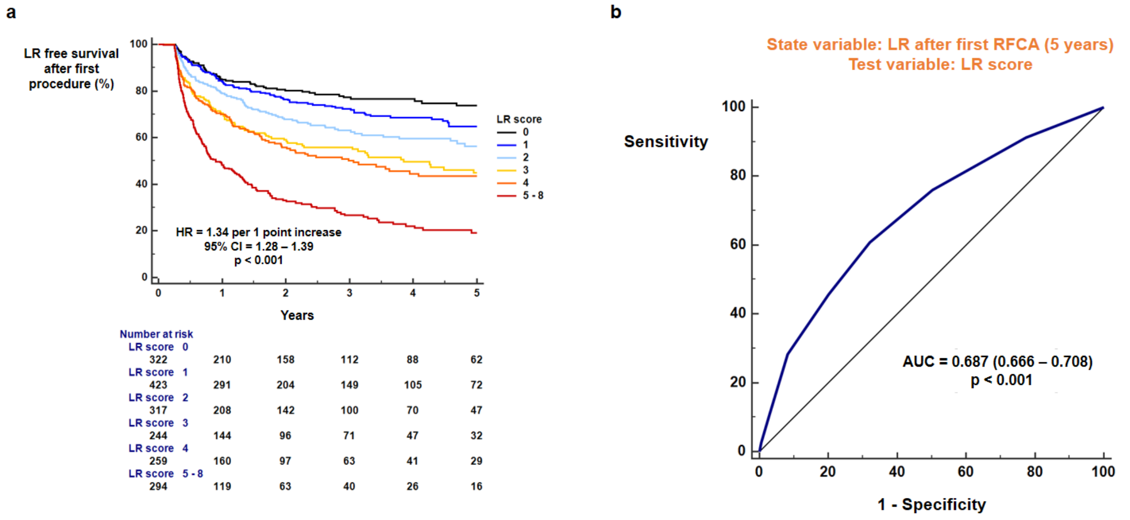

Supplementary Figure S4. Predictive value of the LR score for LR following first-time RFCA.

(a) Risk of LR after first-time RFCA according to LR score.

(b) ROC curve analysis of the LR score for the prediction of LR following first-time RFCA.

AUC: area under curve; HR: hazard ratio; LR: late recurrence; RFCA: radiofrequency catheter ablation; ROC: receiver operating characteristic.

Figure S5

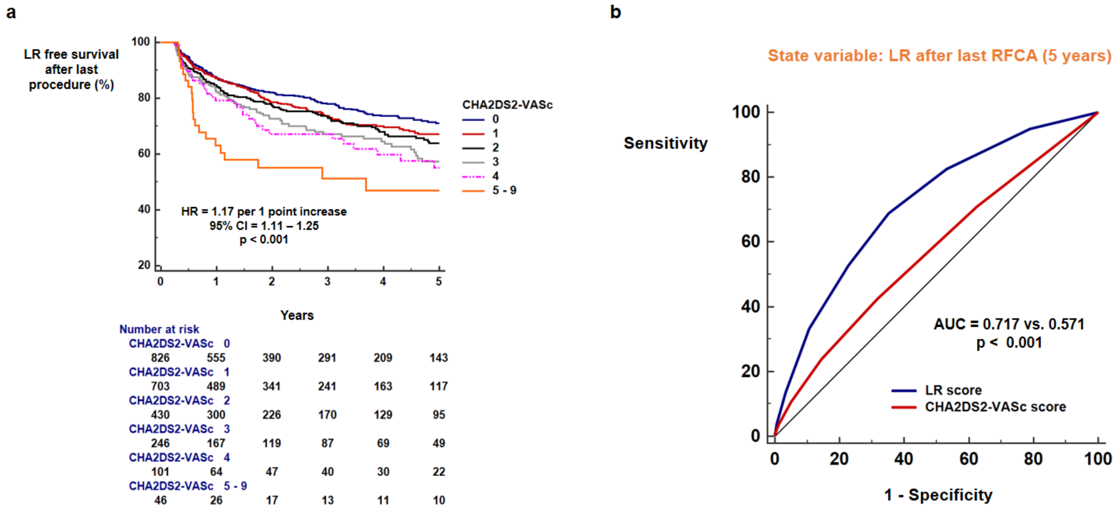

Supplementary Figure S5. Predictive value of CHA_2DS_2-VASc score.

(A) Risk of LR stratified by CHA_2DS_2-VASc score.

(B) ROC curve analysis of the LR score which showed significant predictive value. However, LR score outperformed CHA_2DS_2-VASc score.

AUC: area under curve; CI: confidence interval; HR: hazard ratio; LR: late recurrence; RFCA: radiofrequency catheter ablation; ROC: receiver operating characteristic.
